# Correction: Shedding dynamics of a DNA virus population during acute and long-term persistent infection

**DOI:** 10.1371/journal.ppat.1014004

**Published:** 2026-03-02

**Authors:** Sylvain Blois, Benjamin M. Goetz, Anik Mojumder, Christopher S. Sullivan

The raw data underlying the graphs presented in Fig 1 and S1 were not originally provided with this article [1]. Additionally, while the raw sequencing data and scripts that generated the graphs for Figs 2-10, S2-S8 were originally provided, the analyzed data giving rise to the graphs were not included. The authors have provided these data as S1 File.

With this Correction, all relevant data are now provided in the below Supporting Information file.

## Supporting information

S1 File Individual-level data underlying Figs 1–10 and Figs S1-S8. (XLSX)
