# Supplementary material for: Shedding dynamics of a DNA virus population during acute and long-term persistent infection
Source: PLoS Pathog. 2025 May 23;21(5):e1013083. doi: 10.1371/journal.ppat.1013083 (PMC12136464; doi:10.1371/journal.ppat.1013083)
Supplement: S1 Fig — Virus replication curves were plotted. A. NMuMG cells infected with WT muPyV (red) or barcoded muPyV (blue) at M.O.I. of 5. B. RPTEC infected with WT BKPyV (blue) or either of two different barcoded BKPyV libraries (red or green) at M.O.I. of 1. Cell-associated virus (muPyV) and supernatant (BKPyV) were harvested at multiple time post-infection (p.i.), in duplicate, and virus concentration was determined by immunofluorescence microscopy for VP1 viral proteins. This analysis shows similar kinetics of infectious virus production and confirms no obvious defect in virus replication caused by the barcode inserts. (PDF) [file ppat.1013083.s001.pdf]

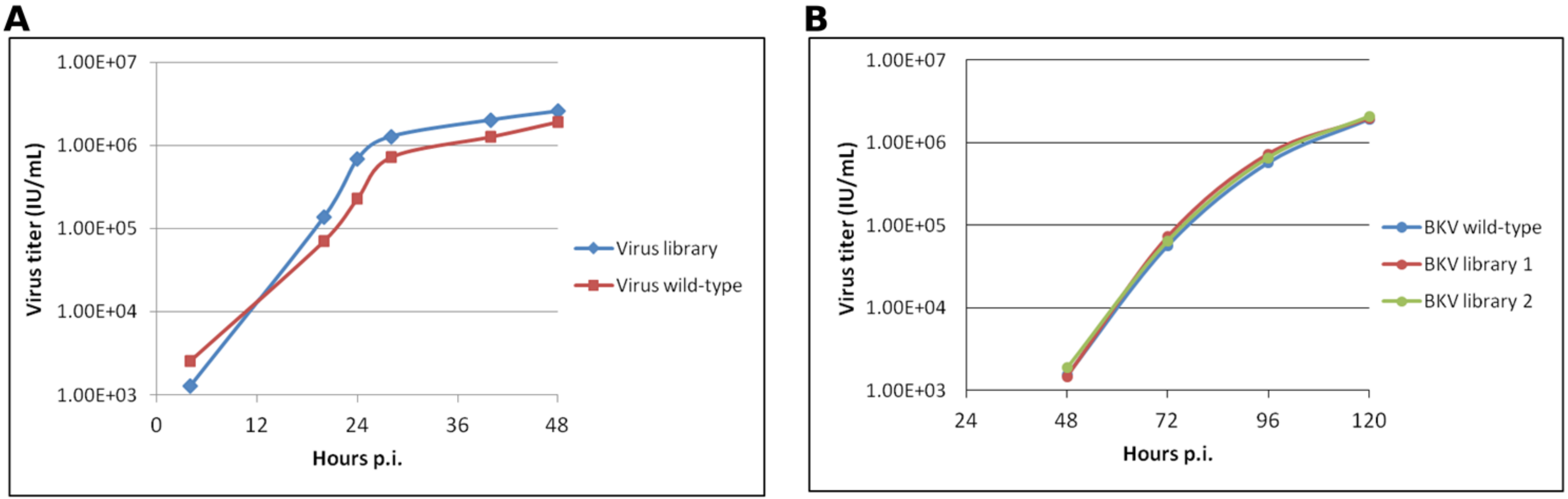

S1 Fig. Barcoded polyomaviruses replicate infectious virus similar to wildtype virus. Virus replication curves were plotted. A. NMuMG cells infected with WT muPyV (red) or barcoded muPyV (blue) at M.O.I. of 5. B. RPTEC infected with WT BKPyV (blue) or either of two different barcoded BKPyV libraries (red or green) at M.O.I. of 1. Cell-associated virus (muPyV) and supernatant (BKPyV) were harvested at multiple time post-infection (p.i.), in duplicate, and virus concentration was determined by immunofluorescence microscopy for VP1 viral proteins. This analysis shows similar kinetics of infectious virus production and confirms no obvious defect in virus replication caused by the barcode inserts.
