# Supplementary material for: Shedding dynamics of a DNA virus population during acute and long-term persistent infection
Source: PLoS Pathog. 2025 May 23;21(5):e1013083. doi: 10.1371/journal.ppat.1013083 (PMC12136464; doi:10.1371/journal.ppat.1013083)

S2 Fig. Ridge plots of the 10 most abundant barcodes. A. Shown in color is the abundance of each of the top 10most shed barcodes in urine for each mouse (“top 10” determined by the greatest amount of a barcode shed at any single time point). The gray shaded area represents total bulk shed viral DNA at a particular time point post-infection. The height of an individual peak on the vertical axis correlates to the relative linear abundance of each barcode. The horizontal axis corresponds to different timepoints post-infection. B. Shows the sum total of the top 10 individual abundant shed barcodes for each mouse in gold (Note: this panel is identical to Fig. 7).

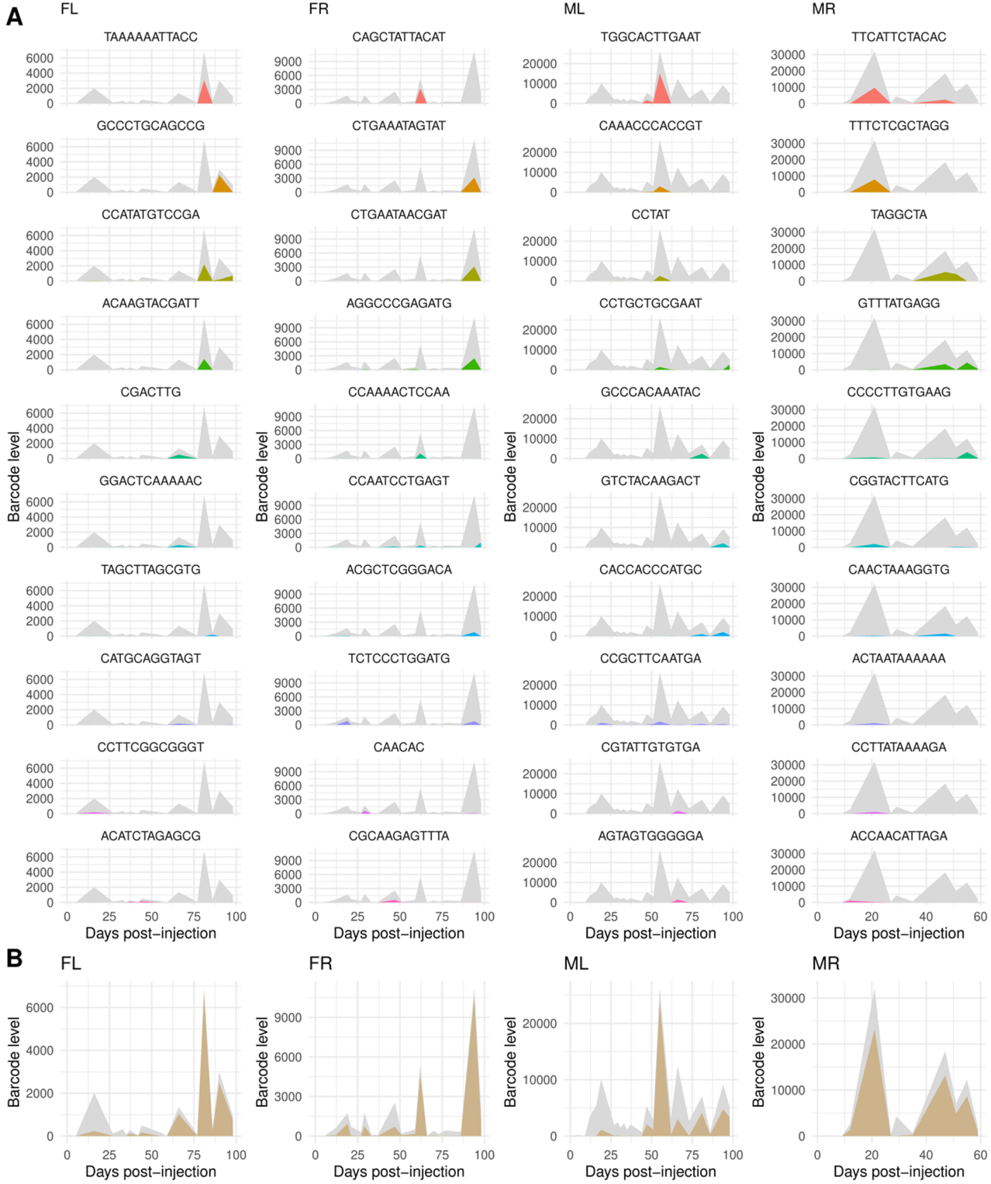

Supplement: S2 Fig — A. Shown in color is the abundance of each of the top 10 most shed barcodes in urine for each mouse (“top 10” determined by the greatest amount of a barcode shed at any single time point). The gray shaded area represents total bulk shed viral DNA at a particular time point post-infection. The height of an individual peak on the vertical axis correlates to the relative linear abundance of each barcode. The horizontal axis corresponds to different timepoints post-infection. B. Shows the sum total of the top 10 individual abundant shed barcodes for each mouse in gold (Note: this panel is identical to Fig 7). (PDF) [file ppat.1013083.s002.pdf]
