# Supplementary material for: Shedding dynamics of a DNA virus population during acute and long-term persistent infection
Source: PLoS Pathog. 2025 May 23;21(5):e1013083. doi: 10.1371/journal.ppat.1013083 (PMC12136464; doi:10.1371/journal.ppat.1013083)
Supplement: S3 Fig — A. GC content for the bulk of all barcodes shed at each time point. B. GC content for the top 10 most shed barcodes for each mouse (“top 10” determined by the greatest amount of a barcode shed at any single time point). Neither panel shows overt trends towards altered nucleotide composition. (PDF) [file ppat.1013083.s003.pdf]

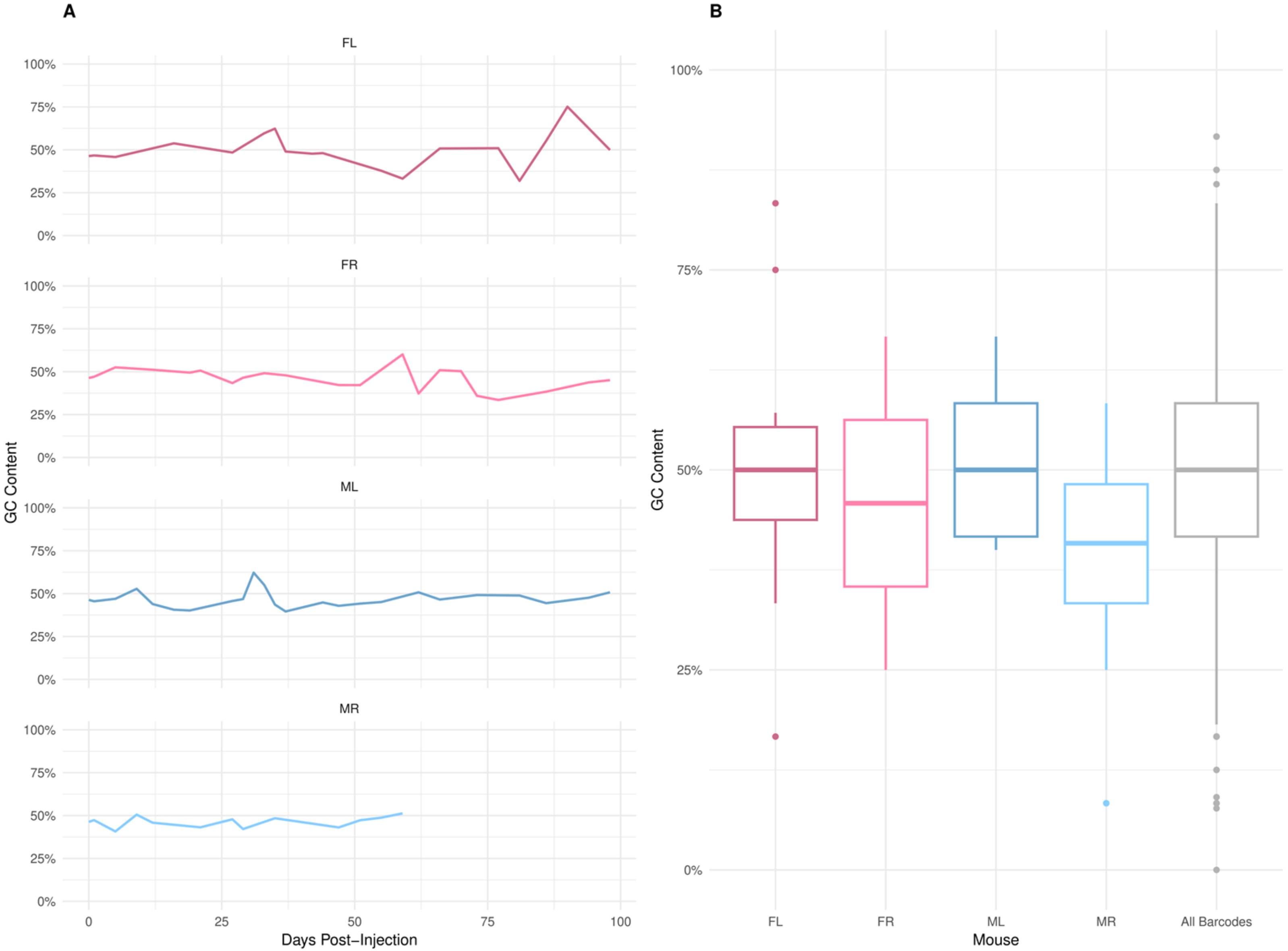

S3 Fig. Mean GC content of shed barcodes does not change substantially during the course of infection. A. GC content for the bulk of all barcodes shed at each time point. B. GC content for the top 10 most shed barcodes for each mouse ("top 10" determined by the greatest amount of a barcode shed at any single time point). Neither panel shows overt trends towards altered nucleotide composition.
