# Supplementary material for: Shedding dynamics of a DNA virus population during acute and long-term persistent infection
Source: PLoS Pathog. 2025 May 23;21(5):e1013083. doi: 10.1371/journal.ppat.1013083 (PMC12136464; doi:10.1371/journal.ppat.1013083)

S4 Fig. Length of top 10 most abundant shed barcodes for each mouse. The length of the barcodes for the top 10 most abundant shed barcodes ("top 10" determined by the greatest amount of a barcode shed at any single time point). Most barcodes retain an insert length of 12 nucleotides, as expected from the library design.

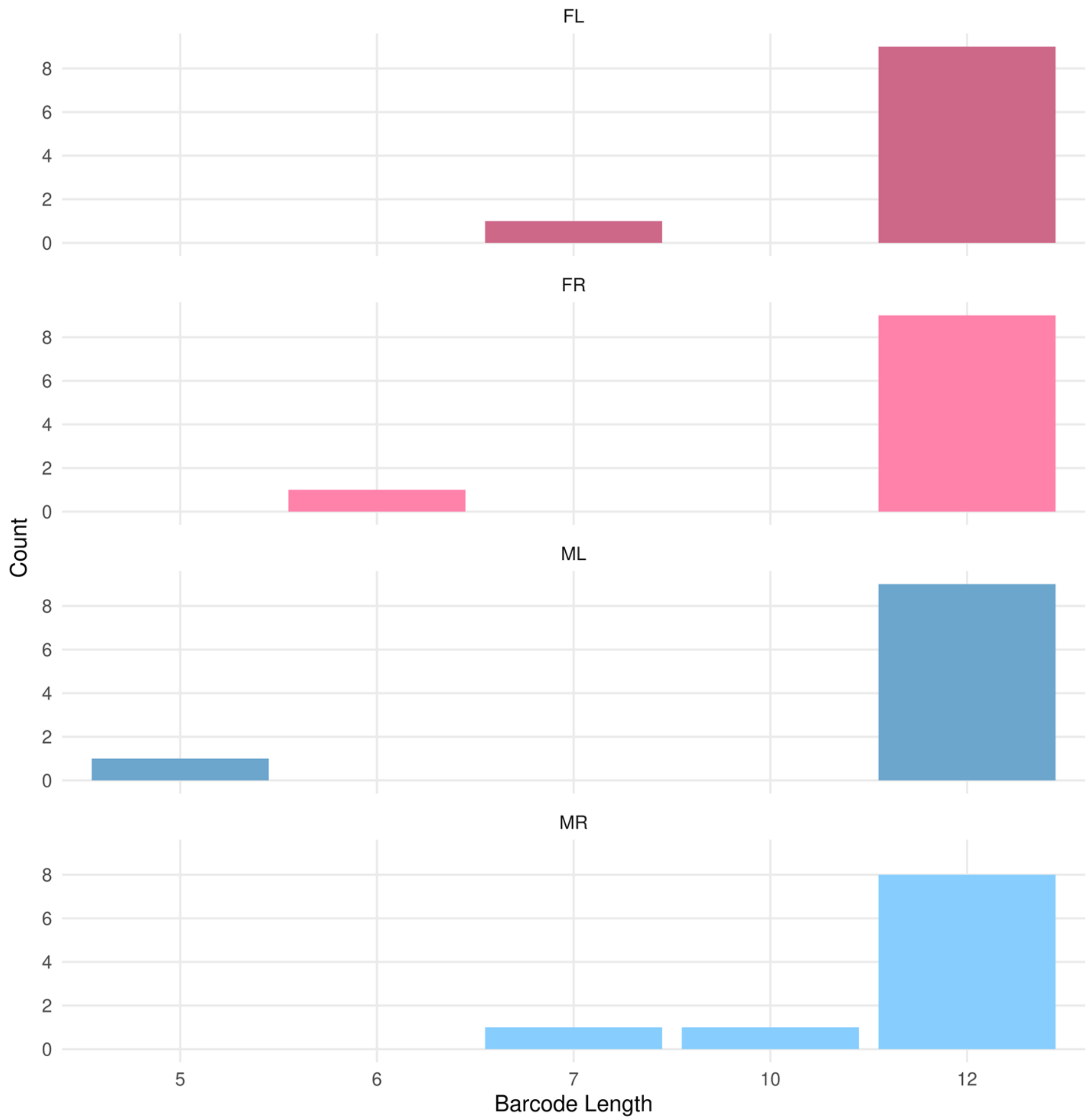

Supplement: S4 Fig — The length of the barcodes for the top 10 most abundant shed barcodes (“top 10” determined by the greatest amount of a barcode shed at any single time point). Most barcodes retain an insert length of 12 nucleotides, as expected from the library design. (PDF) [file ppat.1013083.s004.pdf]
