# Supplementary material for: Shedding dynamics of a DNA virus population during acute and long-term persistent infection
Source: PLoS Pathog. 2025 May 23;21(5):e1013083. doi: 10.1371/journal.ppat.1013083 (PMC12136464; doi:10.1371/journal.ppat.1013083)

S5 Fig. Total amounts of muPyV DNA in organs. Plotted are muPyV genomes equivalent determined by qPCR and normalized per µg of total DNA purified from organs or per µl of whole blood.

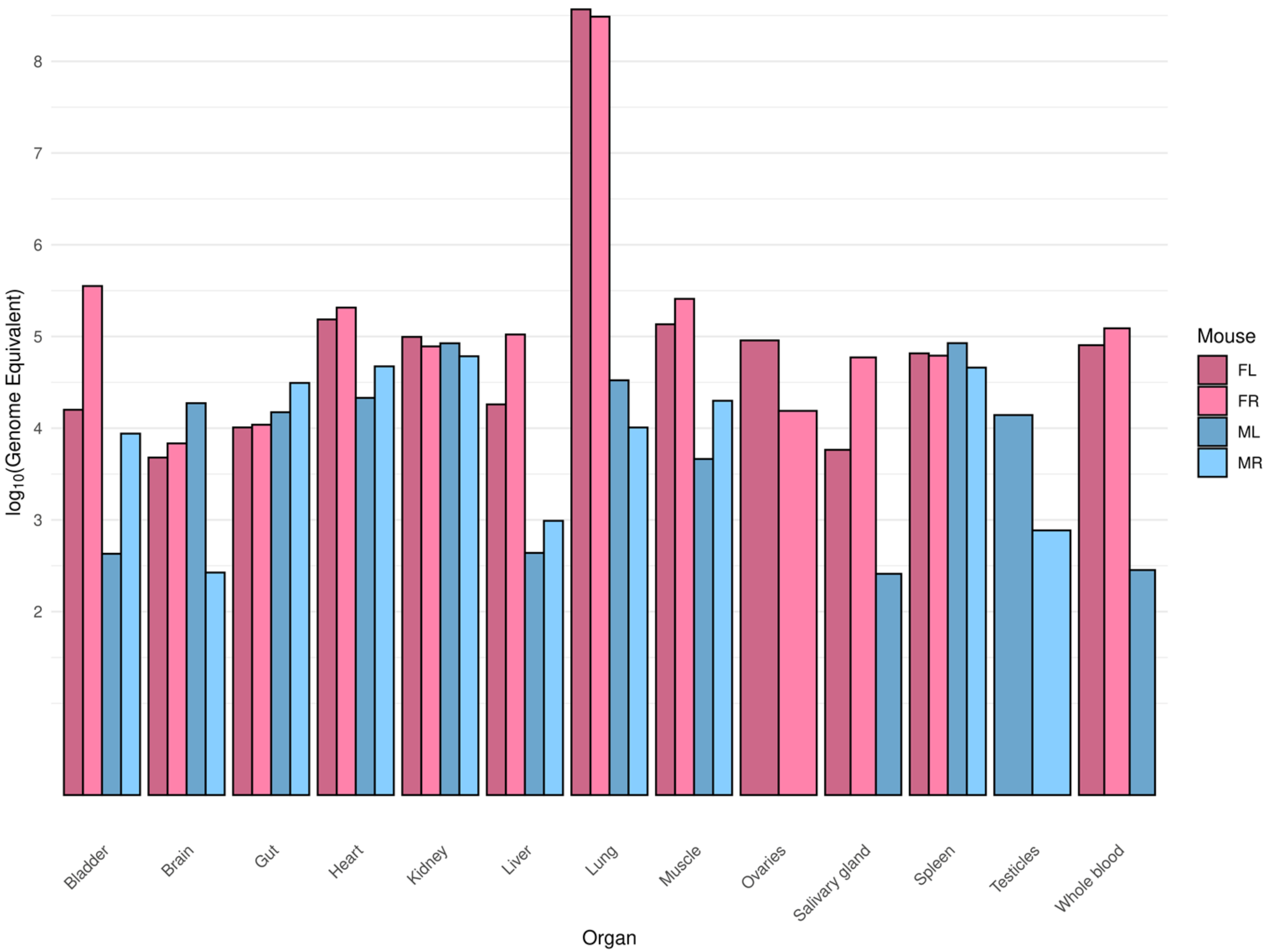

Supplement: S5 Fig — Plotted are muPyV genome equivalents determined by qPCR and normalized per µg of total DNA purified from organs or per µl of whole blood. (PDF) [file ppat.1013083.s005.pdf]
