# Supplementary material for: Shedding dynamics of a DNA virus population during acute and long-term persistent infection
Source: PLoS Pathog. 2025 May 23;21(5):e1013083. doi: 10.1371/journal.ppat.1013083 (PMC12136464; doi:10.1371/journal.ppat.1013083)
Supplement: S6 Fig — The y-axis represents the −log10 transformed p-values from Fisher’s exact test, indicating the strength of association between barcode presence in urine and tissue. Asterisks denote significance levels: *p<0.05, **p<0.01, ***p<0.001, ****p<0.0001, and *****p<0.00001. (PDF) [file ppat.1013083.s006.pdf]

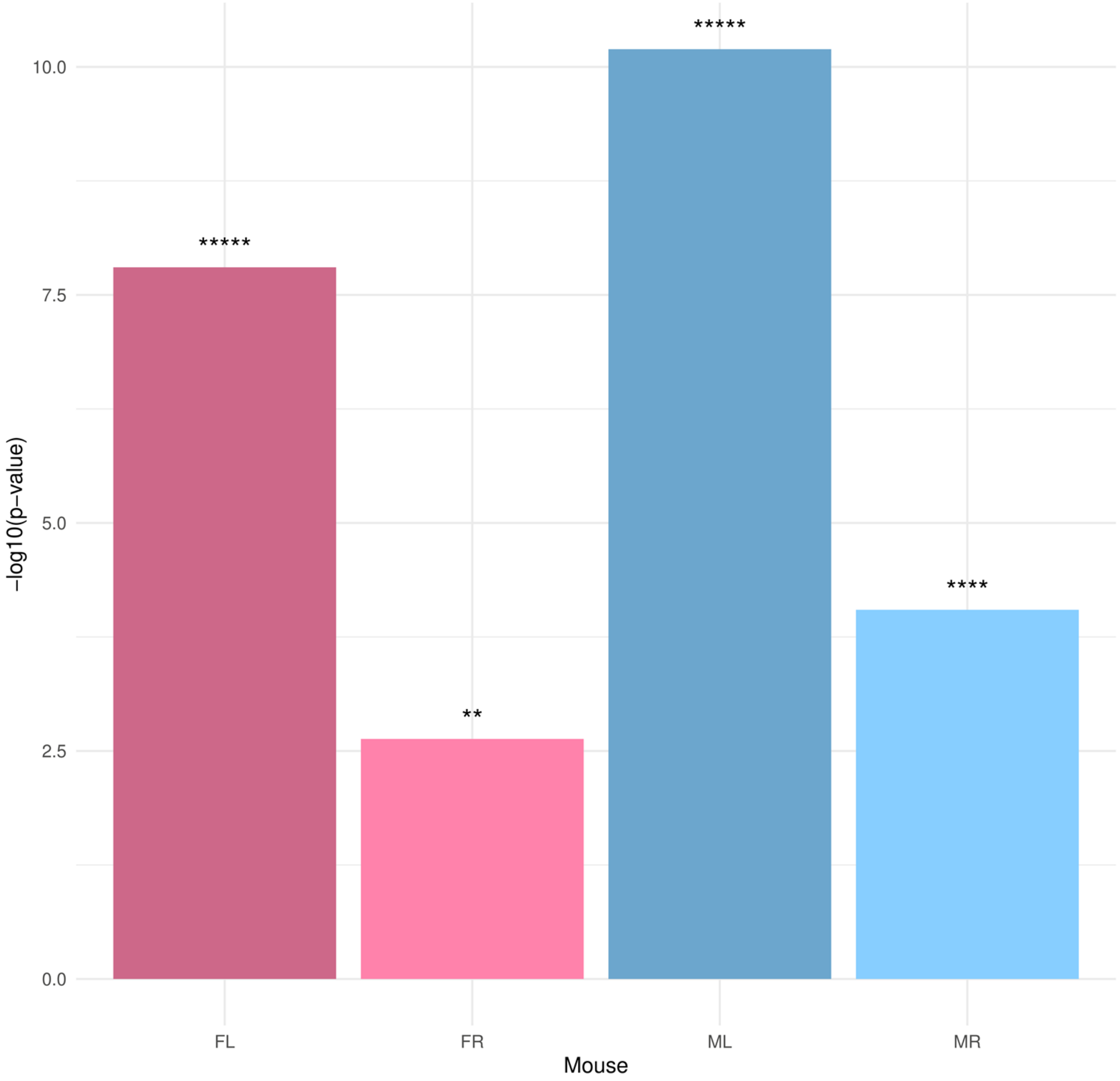

S6 Fig. Statistical significance of barcode overlap between the top 5% of barcodes in urine and tissue samples across animals. The y-axis represents the −log10 transformed p-values from Fisher's exact test, indicating the strength of association between barcode presence in urine and tissue. Asterisks denote significance levels: *p<0.05, **p<0.01, ***p<0.001, ****p<0.0001, and *****p<0.00001.
