# Supplementary material for: Shedding dynamics of a DNA virus population during acute and long-term persistent infection
Source: PLoS Pathog. 2025 May 23;21(5):e1013083. doi: 10.1371/journal.ppat.1013083 (PMC12136464; doi:10.1371/journal.ppat.1013083)

S7 Fig. GC Content of top 10 most abundant barcodes in tissues in each animal. Shown is the GC content of the top 10 most abundant barcodes with abundance determined as the sum total of genome equivalents for each barcode in all tissues assayed for that animal ("top 10" determined by the greatest amount of a barcode shed at any single time point).

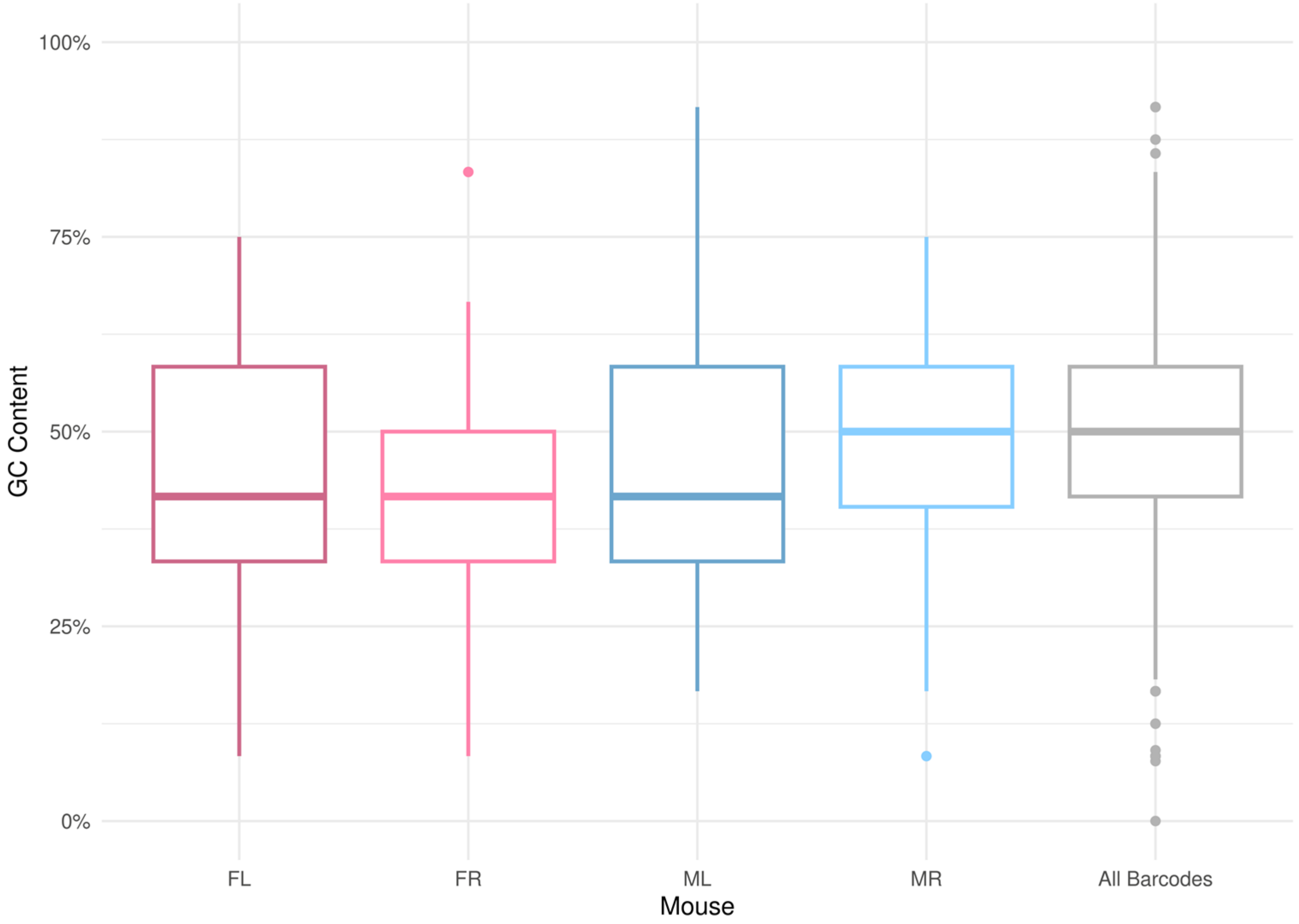

Supplement: S7 Fig — Shown is the GC content of the top 10 most abundant barcodes with abundance determined as the sum total of genome equivalents for each barcode in all tissues assayed for that animal (“top 10” determined by the greatest amount of a barcode shed at any single time point). (PDF) [file ppat.1013083.s007.pdf]
