# Supplementary material for: Shedding dynamics of a DNA virus population during acute and long-term persistent infection
Source: PLoS Pathog. 2025 May 23;21(5):e1013083. doi: 10.1371/journal.ppat.1013083 (PMC12136464; doi:10.1371/journal.ppat.1013083)
Supplement: S8 Fig — Shown are the top 10 most abundant barcodes detected in each organ of a given mouse and their rank in urine that was collected on the final day before sacrifice (“top 10” determined by the greatest amount of a barcode in any tissue for an individual mouse). In 3 of the 4 mice, abundant barcodes in the kidney are clearly also more abundant in urine. No other organ or tissue displayed such a strong signature consistent with shed viruses deriving from the kidney. (PDF) [file ppat.1013083.s008.pdf]

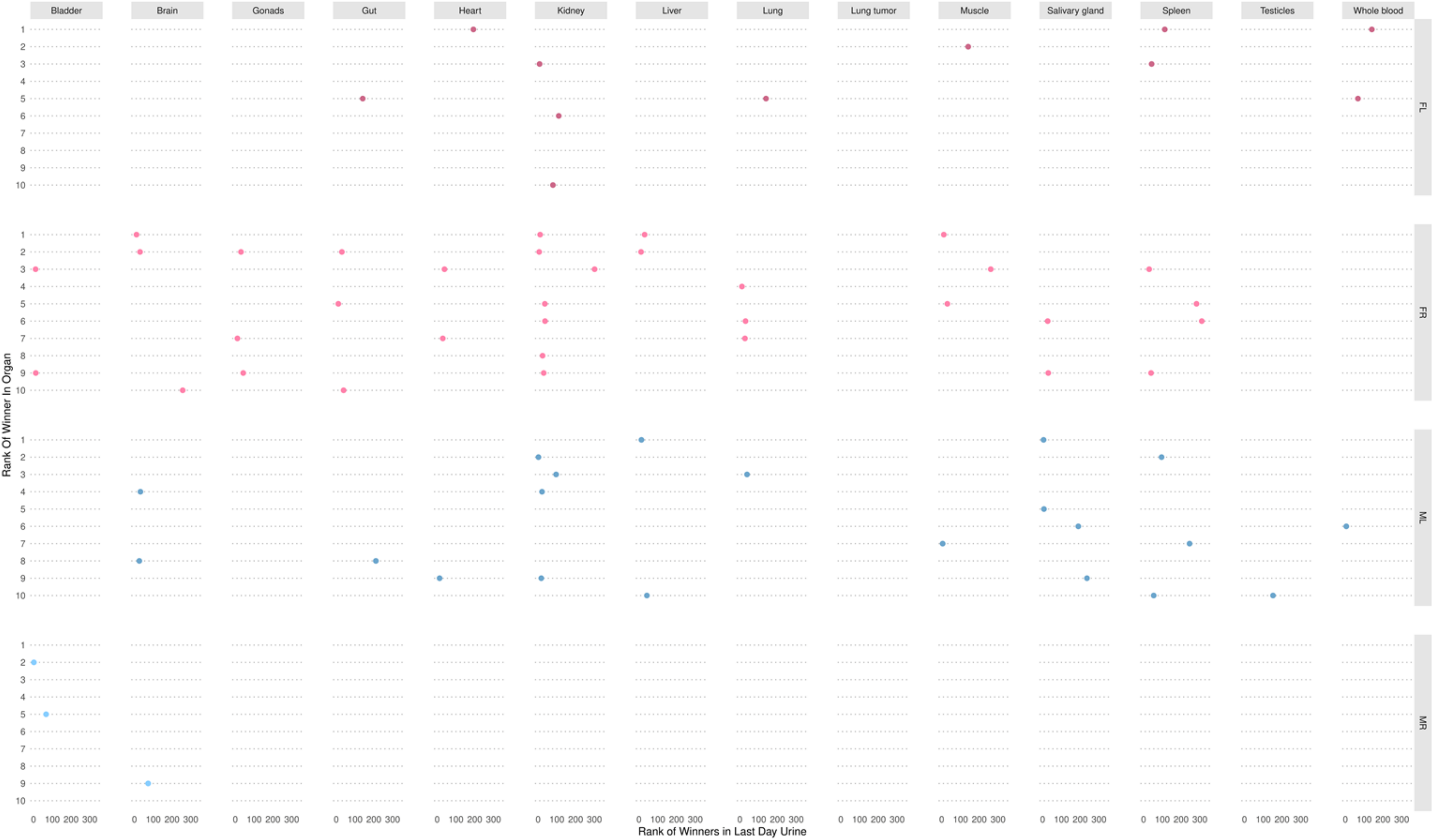

S8 Fig. Abundant barcodes in kidney are more abundantly shed in urine. Shown are the top 10 most abundant barcodes detected in each organ of a given mouse and their rank in urine that was collected on the final day before sacrifice ("top 10" determined by the greatest amount of a barcode in any tissue for an individual mouse). In 3 of the 4 mice, abundant barcodes in the kidney are clearly also more abundant in urine. No other organ or tissue displayed such a strong signature consistent with shed viruses deriving from the kidney.
