# Supplementary material for: Shedding dynamics of a DNA virus population during acute and long-term persistent infection
Source: PLoS Pathog. 2025 May 23;21(5):e1013083. doi: 10.1371/journal.ppat.1013083 (PMC12136464; doi:10.1371/journal.ppat.1013083)
Supplement: S1 Table — (PDF) [file ppat.1013083.s009.pdf]

**S1 Table: Virus stocks titer (IU/mL) as determined by immunofluorescence assay.**

| | **PTA muPyV** | **Dunlop BKPyV** |
|---|---|---|
| Virus wild-type | $7.14 \times 10^6$ | $2.22 \times 10^6$ |
| Virus library 1 | $2.50 \times 10^6$ | $3.08 \times 10^6$ |
| Virus library 2 | N/A | $3.24 \times 10^6$ |
