# Supplementary material for: Shedding dynamics of a DNA virus population during acute and long-term persistent infection
Source: PLoS Pathog. 2025 May 23;21(5):e1013083. doi: 10.1371/journal.ppat.1013083 (PMC12136464; doi:10.1371/journal.ppat.1013083)
Supplement: S2 Table — (PDF) [file ppat.1013083.s010.pdf]

S2 Table: Urine from infected mice contains infectious muPyV as shown by 100% CPE on NMuMG cells

		Urine day 18 p.i.		Urine day 19 p.i.		Urine day 50 p.i.		Urine day 114 p.i.		Urine day 127 p.i.		Urine day 169 p.i.	
	Gender	Genomes eq / ul in urine used to infect cells	Days to reach 100% CPE	Genomes eq / ul in urine used to infect cells	Days to reach 100% CPE	Genomes eq / ul in urine used to infect cells	Days to reach 100% CPE	Genomes eq / ul in urine used to infect cells	Days to reach 100% CPE	Genomes eq / ul in urine used to infect cells	Days to reach 100% CPE	Genomes eq / ul in urine used to infect cells	Days to reach 100% CPE
**Pool of urine**	N/A	3.06E+04	9	1.35E+05	9	7.87E+04	14	N/A	N/A	N/A	N/A	N/A	N/A
**Mouse 1**	male	N/A	N/A	N/A	N/A	N/A	N/A	9.30E+03	16	1.83E+04	15	4.82E+04	19
**Mouse 2**	male	N/A	N/A	N/A	N/A	N/A	N/A	3.42E+04	17	8.84E+03	18	5.91E+04	19
**Mouse 4**	male	N/A	N/A	N/A	N/A	N/A	N/A	2.17E+03	18	5.30E+03	18	6.67E+03	16
**Mouse 5**	female	N/A	N/A	N/A	N/A	N/A	N/A	3.29E+03	18	4.09E+03	17	2.27E+04	19

Controls: NMuMG cells treated with urine filtrates from mock infected mice were negative at all tested time points
At CPE 100%, muPyV vDNA reached at least 1E+08 genome equivalent per µl of supernatant, as determined by qPCR.
