# Supplementary material for: Shedding dynamics of a DNA virus population during acute and long-term persistent infection
Source: PLoS Pathog. 2025 May 23;21(5):e1013083. doi: 10.1371/journal.ppat.1013083 (PMC12136464; doi:10.1371/journal.ppat.1013083)
Supplement: S3 Table — (PDF) [file ppat.1013083.s011.pdf]

S3 Table - Median rank of top 10 most abundant in urine or tissue for each mouse.

Animal	Urine	Tissue
FL	1180.5	884
FR	456	848
ML	651.5	292.5
MR	1266.5	1312.5
