# Supplementary material for: Shedding dynamics of a DNA virus population during acute and long-term persistent infection
Source: PLoS Pathog. 2025 May 23;21(5):e1013083. doi: 10.1371/journal.ppat.1013083 (PMC12136464; doi:10.1371/journal.ppat.1013083)
Supplement: S4 Table — (PDF) [file ppat.1013083.s012.pdf]

S4 Table: Mutations in muPyV genomes found in female mice's lung and tumor.

Mouse	Tissue	Clone	Barcode	Mutation				
				NCCR-VP coding region*	VP1 coding region**	NCCR*	Middle and small T antigens coding region**	Large T-antigen coding region **
FL	Tumor	LTFL5	CCACCCTATAAA	In frame deletion 2415-3466 (1052bp)	Point mutation: Leu38 CTG>TTG	-	-	-
	Lung	LUFL1	AAGTACGGGCGC	In frame deletion 2920-3544 (625bp)	-	Point mutations: 3686 G>T 3788 C>A	Frameshift deletion: Trp88 CCA>-CA	Substitution: Ala50Val GCC>GTC
		LUFL5	CGGAAGGTCATG	In frame deletion 2920-3544 (625bp)	-	Point mutation: 3686 G>T	-	-
FR	Lung	LUFR17	CAGCCCATCTAA	In frame deletion 2037-3540 (1504bp)	-	-	-	-

*Nucleotide and **amino acid positions is referred to the muPyV strain PTA genome (GenBank No U27812).
"-" stands for no mutation found.
