# Supplementary material for: Shedding dynamics of a DNA virus population during acute and long-term persistent infection
Source: PLoS Pathog. 2025 May 23;21(5):e1013083. doi: 10.1371/journal.ppat.1013083 (PMC12136464; doi:10.1371/journal.ppat.1013083)
Supplement: S5 Table — (PDF) [file ppat.1013083.s013.pdf]

S5 Table: Staggered indexing primers.

	Name	Index adapter	i5 or i7 index	Index adapter	Spacer	Annealing sequence
**i5 index forward primers**	>NGS_FwdIN2-i5-U6-F3-D501	AATGATACGGCGACCACCGAGATCTACAC	TATAGCCT	ACACTCTTTCCCTACACGACGCTCTTCCGATCT	CAG	GACTGTATTTCCTGGAAATTAATGTT
	>NGS_FwdIN2-i5-U6-F3-D502	AATGATACGGCGACCACCGAGATCTACAC	ATAGAGGC	ACACTCTTTCCCTACACGACGCTCTTCCGATCT	CAG	GACTGTATTTCCTGGAAATTAATGTT
	>NGS_FwdIN2-i5-U6-F2-D503	AATGATACGGCGACCACCGAGATCTACAC	CCTATCCT	ACACTCTTTCCCTACACGACGCTCTTCCGATCT	G	GACTGTATTTCCTGGAAATTAATGTT
	>NGS_FwdIN2-i5-U6-F2-D504	AATGATACGGCGACCACCGAGATCTACAC	GGCTCTGA	ACACTCTTTCCCTACACGACGCTCTTCCGATCT	G	GACTGTATTTCCTGGAAATTAATGTT
	>NGS_FwdIN2-i5-U6-F1-D505	AATGATACGGCGACCACCGAGATCTACAC	AGGCGAAG	ACACTCTTTCCCTACACGACGCTCTTCCGATCT		GACTGTATTTCCTGGAAATTAATGTT
	>NGS_FwdIN2-i5-U6-F1-D506	AATGATACGGCGACCACCGAGATCTACAC	TAATCTTA	ACACTCTTTCCCTACACGACGCTCTTCCGATCT		GACTGTATTTCCTGGAAATTAATGTT
	>NGS_FwdIN2-i5-U6-F4-D507	AATGATACGGCGACCACCGAGATCTACAC	CAGGACGT	ACACTCTTTCCCTACACGACGCTCTTCCGATCT	GCAC	GACTGTATTTCCTGGAAATTAATGTT
	>NGS_FwdIN2-i5-U6-F4-D508	AATGATACGGCGACCACCGAGATCTACAC	GTACTGAC	ACACTCTTTCCCTACACGACGCTCTTCCGATCT	GCAC	GACTGTATTTCCTGGAAATTAATGTT
	>NGS_FwdIN2-i5-U6-F5-D509	AATGATACGGCGACCACCGAGATCTACAC	TTCGGATG	ACACTCTTTCCCTACACGACGCTCTTCCGATCT	AGCAC	GACTGTATTTCCTGGAAATTAATGTT
	>NGS_FwdIN2-i5-U6-F5-D510	AATGATACGGCGACCACCGAGATCTACAC	ACTCATAA	ACACTCTTTCCCTACACGACGCTCTTCCGATCT	AGCAC	GACTGTATTTCCTGGAAATTAATGTT
**i7 index reverse primers**	>NGS_Rev-i7-U6-R-D701	CAAGCAGAAGACGGCATACGAGAT	ATTACTCG	GTGACTGGAGTTCAGACGTGTGCTCTTCCGATCT		GAATATAGCTGAATACACAGTTTATTC
	>NGS_Rev-i7-U6-R-D702	CAAGCAGAAGACGGCATACGAGAT	TCCGGAGA	GTGACTGGAGTTCAGACGTGTGCTCTTCCGATCT		GAATATAGCTGAATACACAGTTTATTC
	>NGS_Rev-i7-U6-R-D703	CAAGCAGAAGACGGCATACGAGAT	CGCTCATT	GTGACTGGAGTTCAGACGTGTGCTCTTCCGATCT		GAATATAGCTGAATACACAGTTTATTC
	>NGS_Rev-i7-U6-R-D704	CAAGCAGAAGACGGCATACGAGAT	GAGATTCC	GTGACTGGAGTTCAGACGTGTGCTCTTCCGATCT		GAATATAGCTGAATACACAGTTTATTC
	>NGS_Rev-i7-U6-R-D705	CAAGCAGAAGACGGCATACGAGAT	ATTCAGAA	GTGACTGGAGTTCAGACGTGTGCTCTTCCGATCT		GAATATAGCTGAATACACAGTTTATTC
	>NGS_Rev-i7-U6-R-D706	CAAGCAGAAGACGGCATACGAGAT	GAATTCGT	GTGACTGGAGTTCAGACGTGTGCTCTTCCGATCT		GAATATAGCTGAATACACAGTTTATTC
	>NGS_Rev-i7-U6-R-D707	CAAGCAGAAGACGGCATACGAGAT	CTGAAGCT	GTGACTGGAGTTCAGACGTGTGCTCTTCCGATCT		GAATATAGCTGAATACACAGTTTATTC
	>NGS_Rev-i7-U6-R-D708	CAAGCAGAAGACGGCATACGAGAT	TAATGCGC	GTGACTGGAGTTCAGACGTGTGCTCTTCCGATCT		GAATATAGCTGAATACACAGTTTATTC
	>NGS_Rev-i7-U6-R-D709	CAAGCAGAAGACGGCATACGAGAT	CGGCTATG	GTGACTGGAGTTCAGACGTGTGCTCTTCCGATCT		GAATATAGCTGAATACACAGTTTATTC
	>NGS_Rev-i7-U6-R-D710	CAAGCAGAAGACGGCATACGAGAT	TCCGCGAA	GTGACTGGAGTTCAGACGTGTGCTCTTCCGATCT		GAATATAGCTGAATACACAGTTTATTC
	>NGS_Rev-i7-U6-R-D711	CAAGCAGAAGACGGCATACGAGAT	TCTCGCGC	GTGACTGGAGTTCAGACGTGTGCTCTTCCGATCT		GAATATAGCTGAATACACAGTTTATTC
	>NGS_Rev-i7-U6-R-D712	CAAGCAGAAGACGGCATACGAGAT	AGCGATAG	GTGACTGGAGTTCAGACGTGTGCTCTTCCGATCT		GAATATAGCTGAATACACAGTTTATTC
